# Demographic characteristics, clinical and laboratory features, and the distribution of pathogenic variants in the *CFTR* gene in the Cypriot cystic fibrosis (CF) population demonstrate the utility of a national CF patient registry

**DOI:** 10.1186/s13023-021-02049-z

**Published:** 2021-10-02

**Authors:** Panayiotis K. Yiallouros, Andreas Μ. Matthaiou, Pinelopi Anagnostopoulou, Panayiotis Kouis, Malgorzata Libik, Tonia Adamidi, Adonis Eleftheriou, Artemios Demetriou, Phivos Ioannou, George A. Tanteles, Constantina Costi, Pavlos Fanis, Milan Macek, Vassos Neocleous, Leonidas A. Phylactou

**Affiliations:** 1 Respiratory Physiology Laboratory, Medical School, Shacolas Educational Centre for Clinical Medicine, University of Cyprus, 215/6 Palaios Dromos Lefkosias Lemesou, 2029 Aglantzia, Nicosia, Cyprus; 2 Paediatric Pulmonology Unit, Hospital ‘Archbishop Makarios III’, Nicosia, Cyprus; 3 Pulmonology Clinic, Nicosia General Hospital, Nicosia, Cyprus; 4 Pulmonology Clinic, Paphos General Hospital, Paphos, Cyprus; 5 Private Practice, Nicosia, Cyprus; 6 Department of Molecular Genetics, Function and Therapy, The Cyprus Institute of Neurology and Genetics, Nicosia, Cyprus; 7 Cyprus School of Molecular Medicine, The Cyprus Institute of Neurology and Genetics, Nicosia, Cyprus; 8 Department of Clinical Genetics, The Cyprus Institute of Neurology and Genetics, Nicosia, Cyprus; 9 Department of Biology and Medical Genetics, 2nd Faculty of Medicine and Motol University Hospital, Charles University, Prague, Czechia

**Keywords:** *CFTR* gene, CFTR modulators, Cystic fibrosis, Next-generation sequencing, Patient registry

## Abstract

**Background:**

Specialized clinical care for cystic fibrosis (CF) in Cyprus, a small island country, has been implemented since the 1990s. However, only recently, a national CF patient registry has been established for the systematic recording of patients’ data. In this study, we aim to present data on the epidemiological, genotypic and phenotypic features of CF patients in the country from the most recent data collection in 2019, with particular emphasis on notable rare or unique cases.

**Results:**

Overall, data from 52 patients are presented, 5 of whom have deceased and 13 have been lost to follow-up in previous years. The mean age at diagnosis was 7.2 ± 12.3 years, and the mean age of 34 alive patients by the end of 2019 was 22.6 ± 13.2 years. Patients most commonly presented at diagnosis with acute or persistent respiratory symptoms (46.2%), failure to thrive or malnutrition (40.4%), and dehydration or electrolyte imbalance (32.7%). Sweat chloride levels were diagnostic (above 60 mmol/L) in 81.8% of examined patients. The most common identified mutation was p.Phe508del (F508del) (45.2%), followed by p.Leu346Pro (L346P) (6.7%), a mutation detected solely in individuals of Cypriot descent. The mean BMI and FEV_1_ z-scores were 0.2 ± 1.3 and − 2.1 ± 1.7 across all age groups, respectively, whereas chronic *Pseudomonas aeruginosa* colonization was noted in 26.9% of patients. The majority of patients (74.5%) were eligible to receive at least one of the available CFTR modulator therapies. In 25% of patients we recovered rare or unique genotypic profiles, including the endemic p.Leu346Pro (L346P), the rare CFTR-dup2, the co-segregated c.4200_4201delTG/c.489 + 3A > G, and the polymorphism p.Ser877Ala.

**Conclusions:**

CF patient registries are particularly important in small or isolated populations, such as in Cyprus, with rare or unique disease cases. Their operation is necessary for the optimization of clinical care provided to CF patients, enabling their majority to benefit from evolving advances in precision medicine.

**Supplementary Information:**

The online version contains supplementary material available at 10.1186/s13023-021-02049-z.

## Background

Cystic Fibrosis (CF; MIM: 219700) is a multi-systemic autosomal recessive rare disease caused by pathogenic, ‘CF-causing’, variants (henceforward mutations) in the Cystic Fibrosis Transmembrane Conductance Regulator (CFTR; MIM: 602421; NM_000492.3) gene. More than 2,000 *CFTR* variants have been identified thus far as compiled by the CFTR1 database (www.genet.sickkids.on.ca), with their type and frequency being variable in different populations.

CF patient registries have been in existence for decades in large countries in Europe, Australia, Canada, and the United States, facilitating comparative research in demographic characteristics, *CFTR* mutation distribution, clinical and laboratory features of CF, and based on comprehensive data analysis enable actual or longitudinal assessment of health care delivery in the field of CF and beyond [1–4]. Nonetheless, the establishment of national CF patient registries in relatively ‘small-sized’ countries and their impact on the quality of care in respective CF populations has not received appropriate attention thus far.

In the Republic of Cyprus, a relatively small island nation located in the Eastern Mediterranean with a population of approximately 888 thousand citizens (Statistical Service, Republic of Cyprus, 2019 census), CF care has been implemented since 1997, after the establishment of a tertiary Paediatric Pulmonology Unit in Nicosia. The first comprehensive report in Cyprus published in 2007 [5] determined that the prevalence of CF at birth is significantly lower (1 in 8000 live births) compared to other European countries [6]. This estimate was based on clinically diagnosed cases according to established diagnostic guidelines [7], using typical clinical and laboratory features, including sweat chloride testing (utilizing pilocarpine iontophoresis) [8] and limited *CFTR* genotyping and sequencing [5].

The major CF-causing mutation in European-derived populations, the p.Phe508del (legacy nomenclature F508del), was also found to be the most common CF allele (50%) in the Greek-Cypriot CF population, while the second most common allele was the p.Leu346Pro (L346P) mutation (11.5%), which has been detected only in the Greek-Cypriot population [9]. This missense mutation is associated with an overall ‘milder’ course of the disease in our paediatric CF cohort and is likely due to an ancient founder effect [5]. Nevertheless, at that time a standard CF patient registry was not implemented and standard long-term monitoring of patients had not been available.

Since January 2017, the nation-wide CF patient registry has become operational and started to provide annual Cypriot data to the European Cystic Fibrosis Society Patient Registry (ECFSPR) (www.ecfs.eu/projects/ecfs-patient-registry/annual-reports). Hence, in the last 3 years, we accrued better understanding of demographic, clinical, laboratory and epidemiological characteristics of CF in Cyprus. At the same time, we have initiated academic collaboration with Charles University (Prague) which carried out an international *CFTR* genotyping project in under-tested CF populations, through a research project funded by an unrestricted charitable donation. This collaboration enabled us to sequence the entire *CFTR* gene by massively parallel sequencing, including intra-*CFTR* rearrangement analysis, in order to detect less common or even unique CF allele genotypes, and thus provide better insight into the distribution of variants in 12 cases where one or both CF-causing mutations remained unidentified with Sanger sequencing.

The main aim of this study is to outline the establishment of the CF patient registry in Cyprus and present a comprehensive analysis of the genotypic and phenotypic characteristics of Cypriot CF patients listed in the national registry until the end of 2019, with an emphasis on the clinical characteristics of rare or unique *CFTR* genotypes that are present at a relatively high frequency in our population. We hope that this study will provide a basis for improving diagnostics and clinical management, including the introduction of variant-specific CF therapies, in Cyprus.

## Results

### Demographic characteristics

As of December 31st, 2019, a total of 52 patients (57.7% males) aged 7.2 ± 12.3 years at diagnosis were registered in the national CF patient registry. Of these, 49 had classic CF and 3 CFTR-related disease. Five patients deceased and 13 were lost to follow-up in the last 3 years. The majority (57.7%) of patients were of Greek-Cypriot origin from both parents, while in 7.7% of patients one parent was Greek-Cypriot.

### Clinical presentation at diagnosis

Most commonly, patients presented with acute or persistent respiratory symptoms (46.2%), failure to thrive or malnutrition (40.4%), and dehydration or electrolyte imbalance (32.7%), either isolated or in combination with other manifestations. Four patients (7.7%) presented with meconium ileus, and three of them were operated, shortly after birth. In 6 (11.5%) patients, clinical presentation was not defined, whereas in 48.1% patients, a combination of two or more manifestations led to further clinical and laboratory investigations which led to the diagnosis of CF (Table 1). Dehydration or electrolyte imbalance as initial manifestation was associated with younger age at diagnosis (r = − 0.36, *p* = 0.02), and occurred primarily (82.4%) during the warm period of the year (approximately May–September) (chi-squared test 5.9; *p* = 0.015).

**Table 1:** Clinical presentation of Cypriot CF patients

Clinical presentation	Number of patients	Percentage of patients (%)
Acute or persistent respiratory symptoms	24	46.2
Failure to thrive or malnutrition	21	40.4
Dehydration and or electrolyte imbalance	17	32.7
Steatorrhea or other gastrointestinal symptoms	7	13.5
Meconium ileus or other intestinal obstruction	5	9.6
Prenatal screening (amniocentesis or chorionic villus sampling)	3	5.8
Family history and genotyping	3	5.8
Neonatal screening	2	3.9
Nasal polyposis and/or sinus disease	1	1.9
Unknown presenting symptoms	6	11.5

### CFTR genotype

**Fig. 1:**
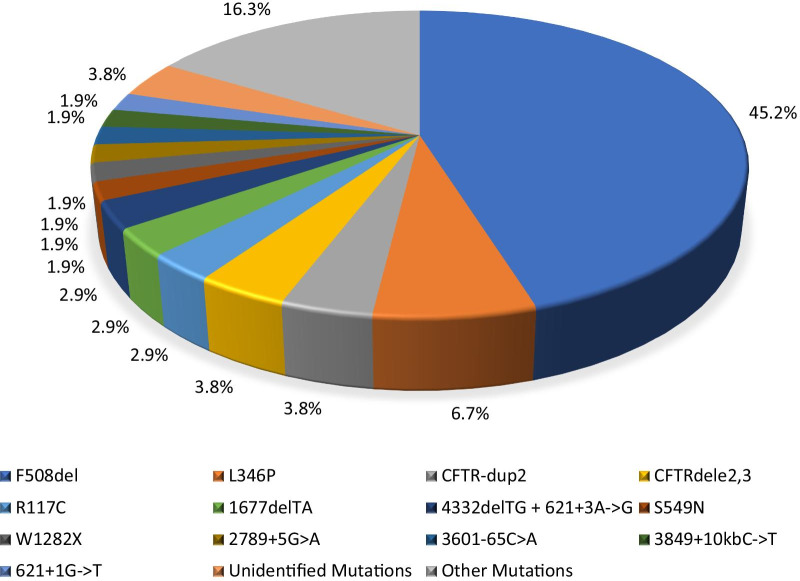
Frequency of *CFTR* mutations in on all CF alleles in the Cypriot population. *CFTR* mutations are presented in legacy nomenclature; the listed variant frequencies are present both in the classical forms of the disease and cases with CFTR-related diseases

We found two CF-causing mutations (in trans on both parental *CFTR* loci) in 49 (94.2%) patients, one mutation in two (3.9%) patients, and no genotypic alterations by the technique applied in one case (1.9%). The three cases that were not fully genotyped are classic CF cases and their samples have not undergone massively parallel sequencing for logistic reasons. The overall population-specific mutation detection rate is 96.2% for the entire cohort of Cypriot cases including the classical form of the disease and the likely CFTR-related diseases (Table 2). The most common mutation was p.Phe508del (F508del) with a frequency of 45.2%, while the second most common allele, the p.Leu346Pro (L346P), was detected in 7 instances (6.7%) of all CF alleles. Another 28 individual *CFTR* mutations were found in patients’ alleles but at very low frequencies (Fig. 1). Two novel CFTR variants (p.Gly178TrpfsX5/c.531dupT and p.Ser877Ala) were found and reported by us to the CFTR1 database (see further). Patients who had CFTR-related diseases had the following *CFTR* alleles: p.Ser877Ala/p.Gly542X (Case 1), 5T(TG11)/p.Lys684SerfsX38 (Case 2), and p.Met348Lys/p.Leu346Pro (Case 3). A sole pathogenic *CFTR* mutation was identified in two cases, whereas in another case no *CFTR* mutations were found at all; in those cases, diagnosis was based on elevated sweat chloride concentrations (> 60 mmol/L) according to diagnostic inclusion criteria.

**Table 2:** Identified *CFTR* mutations

*CFTR* mutation			*CFTR* allelic number	*CFTR* allelic frequency (%)	Number of compound heterozygous/homozygous patients
Legacy name	cDNA name (NM_000492.3)	Protein name			
F508del	c.1521_1523delCTT	p.Phe508del	47	45.2	22/13
L346P	c.1037T > C	p.Leu346Pro	7	6.7	7/0
CFTR-dup2	NA	NA	4	3.8	0/2
CFTRdele2,3	c.54-5940_273 + 10250del21kb	p.Ser18ArgfsX16	4	3.8	4/0
R117C	c.349C > T	p.Arg117Cys	3	2.9	3/0
1677delTA	c.1545_1546delTA	p.Tyr515X	3	2.9	3/0
4332delTG + 621 + 3A- > G	c.4200_4201delTG + c.489 + 3A > G	p.Cys1400X	3	2.9	3/0
S549N	c.1646G > A	p.Ser549Asn	2	1.9	2/0
W1282X	c.3846G > A	p.Trp1282X	2	1.9	2/0
2789 + 5G > A	c.2657 + 5G > A	NA	2	1.9	2/0
3601-65C > A	NA	NA	2	1.9	2/0
3849 + 10kbC- > T	c.3717 + 12191C > T	NA	2	1.9	2/0
621 + 1G- > T	c.489 + 1G > T	NA	2	1.9	2/0
CFTRdele4-11	NA	NA	1	1.0	1/0
D110H	c.328G > C	p.Asp110His	1	1.0	1/0
E379X	c.1135G > T	p.Glu379X	1	1.0	1/0
G542X	c.1624G > T	p.Gly542X	1	1.0	1/0
G551D	c.1652G > A	p.Gly551Asp	1	1.0	1/0
M348K	c.1043T > A	p.Met348Lys	1	1.0	1/0
N1303K	c.3909C > G	p.Asn1303Lys	1	1.0	1/0
N1303K + 3601-65C > A	c.3909C > G	p.Asn1303Lys	1	1.0	1/0
Q1476X	c.4426C > T	p.Gln1476X	1	1.0	1/0
R1066C	c.3196C > T	p.Arg1066Cys	1	1.0	1/0
R117H	c.350G > A	p.Arg117His	1	1.0	1/0
R347P	c.1040G > C	p.Arg347Pro	1	1.0	1/0
2183AA- > G	c.2051_2052delAAinsG	p.Lys684SerfsX38	1	1.0	1/0
4382delA	c.4251delA	p.Glu1418ArgfsX14	1	1.0	1/0
NA	c.2629T > G^a^	p.Ser877Ala^a^	1	1.0	1/0
NA	c.531dupT^a^	p.Gly178TrpfsX5^a^	1	1.0	1/0
5T (TG11)	NA	NA	1	1.0	1/0
Unidentified Mutations	NA	NA	4	3.8	2/1

*NA* not applicable

^a^Novel alleles in this study

### Sweat chloride concentrations

Sweat chloride concentrations were reported in 44 (84.6%) patients, with 18 (34.6%) patients having two serial measurements at least 4 weeks apart. Sweat chloride levels above 60 mmol/L in at least one measurement were found in 36 (81.8%) patients, between 30 and 60 mmol/L in a single and/or recurrent measurement in 9 (20.5%) patients, and below 30 mmol/L in a single and/or recurrent measurement in 3 (6.8%) patients. The sweat chloride concentrations of the three CFTR-related disease cases were 23.5 and 21 mmol/L (Case 1), 54 mmol/L (Case 2), and 19 mmol/L (Case 3).

### Clinical and laboratory characteristics

**Fig. 2:**
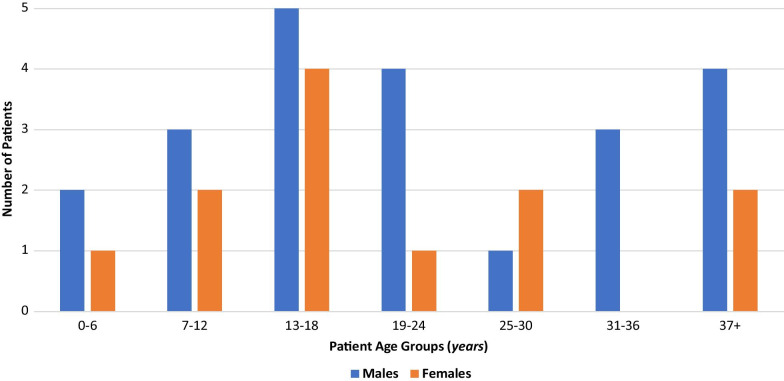
Gender and age distribution of living Cypriot CF patients by the end of 2019

From a total of 34 followed-up patients aged 22.6 ± 13.2 years by the end of 2019 (Fig. 2), 30 (85.3%) patients provided data during years 2018 and 2019.

### Nutrition and lung function

The mean BMI z-score was 0.2 ± 1.3 across all age groups, − 0.1 ± 1.6 in patients aged 2–17 years, and 0.3 ± 0.9 (mean BMI 23 ± 3.8) in adult patients. The mean FEV_1_ z-score in patients who had never had a lung transplant was − 2.1 ± 1.7 across all age groups, − 1.5 ± 1.4 in patients aged 6–17 years, and − 2.5 ± 1.7 in adults.

Overall, 23 (76.7%) patients underwent HRCT chest scan in previous years, and among them 19 (82.6%) patients were found to have bronchiectasis of variable severity. In 13 (43.3%) cases, digital clubbing was observed at physical examination. A 37-year-old male underwent lung transplantation at the age of 27 years in Vienna, Austria.

### Airway microbiology

Twenty-six (86.7%) patients had at least one positive sputum sample or cough swab culture for 8 common pathogens. The most common pathogens were *Haemophilus influenzae* (19, 73.1%), methicillin-sensitive *Staphylococcus aureus* (13, 50%), and *Pseudomonas aeruginosa* (14, 53.9%). Only one (3.9%) patient was found to be positive for *Burkholderia cepacia* complex. Seven patients (26.9%) had chronic *Pseudomonas aeruginosa* colonization.

### Symptomatic treatment

Pancreatic enzyme supplements were taken by 66.7% and multivitamins by 76.7% of patients. The majority of patients were taking recombinant human DNAse (63.3%) and regularly underwent chest physiotherapy (53.3%). Continuous inhaled antibiotic, i.e. *tobramycin*, was prescribed for 9 (30%) patients, including all seven patients with confirmed chronic *Pseudomonas aeruginosa* lung colonization. Six (20%) patients were hospitalized and received intravenous antibiotic therapy on one or more occasions during the years 2018–2019. CFTR modulator therapy was administered in 3 (10%) p.Phe508del (F508del) homozygous patients (see further). Details of the treatment modalities used during 2018 and 2019 appear in Additional file 1: Table S3.

### Complications

Among the 34 followed-up patients, one has developed CF-related diabetes mellitus, chronic pancreatitis, and mild hepatobiliary disease. In previous years, four patients succumbed to severe chest infections and respiratory failure, and one to biliary cirrhosis, portal hypertension, and liver failure. The mean age of death was 26.4 years.

### Eligibility for CFTR modulator therapy

Overall, 47 patients, including 34 followed-up and 13 lost to follow-up, were assessed for their eligibility to receive at least one of the four currently available targeted CFTR modulator therapies. Seventeen (36.2%) patients are eligible to receive one of the three CFTR modulator and potentiator drugs that were available prior to 2020. The recently approved triple combination of *tezacaftor*, *ivacaftor* and *elexacaftor* [10–13] significantly expanded the group of eligible patients and when the age limit for this combinations drops to 2 years of age this number will rise to a total of 31 (66% of the entire CF population). From the patients who are ineligible to receive the triple combination, three fulfil the criteria to take *ivacaftor* and one to take double combination of *tezacaftor* and *ivacaftor*. Thus, a total of 35 (74.5%) patients in our centre are eligible to receive advanced variant-specific therapies. Table 3 shows numbers of eligible patients for treatment with the four CFTR modulator drugs in Cyprus.

**Table 3:** Eligibility for administration of CFTR modulators

CFTR modulator	Indications for use	Number (percentage, %) of currently eligible patients	Number (percentage, %) of futurely^a^ eligible patients
Elexacaftor/Tezacaftor/Ivacaftor	At least one p.Phe508del	23 (48.9%)	8 (17%)
Lumacaftor/Ivacaftor	Two p.Phe508del	9 (19.2%)	0 (0%)
Tezacaftor/Ivacaftor	Two p.Phe508del or at least one *CFTR* mutation responsive to therapy based on clinical and/or in vitro assay data	11 (23.4%)	5 (10.6%)
Ivacaftor	At least one *CFTR* mutation responsive to therapy based on clinical and/or in vitro assay data	7 (14.9%)	0 (0%)

^a^Eligible in the future when indications will include children less than 12 years of age

In 13 of the 52 (25%) patients in Cyprus we recovered rare or unique *CFTR* genotypes. Those rare cases are briefly presented below, while a more detailed description is available in Additional file 1.

### Cases with the p.Leu346Pro (L346P) mutation in compound heterozygosity

The p.Leu346Pro (L346P) mutation has been reported only in seven patients of Cypriot descent, usually presenting in childhood with dehydration or electrolyte imbalance, followed by late-onset lung disease and bronchiectasis in adolescence and early adulthood (Table 4).

**Table 4:** Diagnostic and clinical features of 7 cases with the ‘Greek-Cypriot’ p.Leu346Pro (L346P) mutation in compound heterozygosity

Features	Case 1	Case 2	Case 3	Case 4	Case 5	Case 6	Case 7
Diagnosis	CF	CF	CF	CF	CF	CF	CFTR-RD
Gender	Male	Female	Male	Female	Male	Female	Female
Age (year*s*)	19.7	21	24.3	27.8	32.2	48.5	72.9
Age at diagnosis (years)	0	0.5	1	0.4	5.7	25.2	47.7
Other *CFTR* mutation	p.Phe508del	p.Phe508del	p.Phe508del	p.Tyr515X	p.Phe508del	CFTRdele2,3	p.Met348Lys
Sweat chloride (mmol/L)^a^	75	106	80	103.5	66.5	86	19
Clinical presentation	PS	RS; EI	EI; FTT	EI; FTT; GS	EI; FTT; GS	RS; FTT	FH
Digital clubbing	No	ND	No	ND	Yes	ND	ND
BMI z-score^b^	− 0.2	0.1	1.2	ND	− 0.2	ND	ND
FEV_1_ z-score^c^	− 0.9	− 2.4	− 0.2	ND	− 4.6	ND	ND
Pancreatic insufficiency^d^	Yes	ND	No	ND	Yes	ND	ND
Chronic *P. aeruginosa* col.^e^	ND	ND	ND	ND	Yes	ND	ND
Bronchiectasis^f^	Moderate	ND	No	Moderate	Severe	ND	ND

^a^Mean value (mmol/L) of all performed sweat chloride tests

^b^BMI z-score measured on date of best FEV_1_ z-score measurement in follow-up year

^c^Best FEV_1_ z-score measured in follow-up year

^d^Pancreatic insufficiency defined as pancreatic enzyme supplementation requirement

^e^Chronic pulmonary colonization with *Pseudomonas aeruginosa* defined according to modified Leeds criteria

^f^Bronchiectasis revealed by chest CT scan. *CFTR-RD* CFTR-related disease, *PS* prenatal screening, *RS* respiratory symptoms, *EI* electrolyte imbalance, *FTT* failure to thrive, *GS* gastrointestinal symptoms, *FH* family history, *ND* not defined

### Cases with the complex allele p.Cys1400X with c.489 + 3A > G in cis

Three children were identified with the known p.Cys1400X (4326delTC) in compound heterozygosity with another known *CFTR* mutation. In all three cases, p.Cys1400Ter co-segregates with the rare splicing mutation c.489 + 3A > G (621 + 3A > G), which was previously reported as having varying clinical consequence [14]. According to the CFTR2 database data, it is more likely to be associated with pancreatic sufficiency (www.cftr2.org/mutation/general/621%252B3A-%253EG/), while two of the cases presented in early childhood with respiratory symptoms, electrolyte imbalance and have extensive bronchiectatic changes.

### Cases with homozygosity of the CFTR-dup2 intra-CFTR rearrangement

Two adult male siblings of Greek-Cypriot origin were found to bear a novel duplication of exon 2 on both parental *CFTR* alleles. The first case presented with persistent respiratory symptoms, pancreatic insufficiency and malnutrition at the age of 41 years. Patient had chronic *Pseudomonas aeruginosa* pulmonary colonization and mild bronchiectatic changes, but developed severe hepatic involvement with biliary cirrhosis and eventually hepatic failure that led to his death at the age of 47 years. Other possible causes of cirrhosis and hepatic failure such as viral hepatitis or excess alcohol consumption were excluded.

The second case was diagnosed at the age of 49 years with persistent respiratory and gastrointestinal symptoms since childhood. He was chronically colonized with *Pseudomonas aeruginosa* with moderate bronchiectatic changes, lung emphysema and digital clubbing. He had an enlarged and diffusely hyperechogenic liver, although liver function tests are within normal. Increased plasma amylase and fasting glucose were indicative of chronic pancreatitis and CF-related diabetes mellitus, respectively.

### Cases with compound heterozygosity of the novel p.Ser877Ala variant

A 14-year-old male was diagnosed after presenting with persistent respiratory symptoms, bearing the common p.Gly542X (G542X) mutation and the novel *CFTR* variant p.Ser877Ala, while he had two negative sweat chloride tests (24 and 21 mmol/L). According to the ACMG.net classification this variant is Class 3—variant of unknown significance [15]. His best FEV_1_ z-score was 0.2, and FVC z-score − 0.8, he had several sputum cultures positive for *Pseudomonas aeruginosa*, and localized mild bronchiectatic changes. He was classified as a CFTR-related disease case [16].

## Discussion

In this report, we present a comprehensive description of the demographic, genotypic, clinical and laboratory features of CF and CFTR-related disease patients in Cyprus from the recently established national registry. Furthermore, we characterized rare or unique *CFTR* genotypes in 25% of cases, underscoring the importance of operating national patient registries in small and/or isolated populations. Since the introduction of the first CF patient registry in the United States in 1966, an increasing number of registries have been established all over the world, with most of them operating at a national level [4]. In parallel, the multinational ECFSPR combines contributions from local registries, concentrating data from 48,000 patients across 35 countries in Europe [17]. The national registry of Cyprus was developed in 2017, aiming to systematically record characteristics of CF patients in the country, and longitudinally follow-up their clinical outcomes, in order to optimize specialized healthcare to patients.

Comparing our data with the latest published ECFSPR Annual Data Report of 2017 [17], the nutritional indices (mean BMI z-score) of our patients are among the higher in Europe, and could be included in the top 5 and 2 countries’ values for patients below and above 18 years of age, respectively. In contrast, the mean FEV_1_% predicted in Cyprus is among the 8 countries with the lowest values for children, whereas it is close to the median value of all 34 participating countries for adults. The percentage of our patients proven to be chronically colonized with *Pseudomonas aeruginosa* is also close to the median value of the other European countries.

The broad eligibility criteria of the recently EMA approved triple combination *elexafactor*-*tezacaftor*-*ivacaftor* [11, 12], significantly expanded the group of eligible patients after the age of 12 years for CFTR modulator therapy to up to 66% of the total CF population in Cyprus. In addition, several other patients bear at least one *CFTR* mutation which has been shown to be responsive to either *ivacaftor* [18] or *ivacaftor*-*tezacaftor* combination [13], raising the total percentage of patients who were eligible for any type of CFTR modulator therapy to 75%. However, these orphan medicinal products have a high economic cost and national health authorities need to receive high quality data for their compensation to eligible CF patients. Currently, only 10% of CF patients in Cyprus have been receiving any kind of CFTR modulator therapy.

A neonatal screening program for CF has not been established in the Republic of Cyprus and the majority of patients are diagnosed after presenting with one or more CF-related manifestations [5, 19]. The most common clinical manifestations at presentation are respiratory symptoms, observed in slightly less than half of the cases, followed by failure to thrive or malnutrition. Due to the temperate dry climate of the country, dehydration or electrolyte imbalance is the third most common presenting manifestation, mainly during the warm period of the year from mid-spring to mid-fall, as it has been also reported from other countries with very warm climates [20–23].

We have assessed *CFTR* genotype using commercial assays [24] comprising common *CFTR* mutations in European-derived populations, complemented with limited DNA sequencing, which led to the diagnosis of many cases, but also failed to identify pathogenic mutations in 29% of cases. By using massively parallel sequencing and MLPA technique for intra-*CFTR* rearrangement analysis additional rare CF alleles and large rearrangements in the *CFTR* gene were detected in incompletely genotyped cases, three of which are novel. Despite the lower prevalence of CF in Greek-Cypriots in comparison to the majority of European countries, p.Phe508del (F508del) is the most common mutation (44.4%) on the island, which is compatible with the observed northwest-to-southeast gradient in Europe [5]. Quite interestingly, we recovered rare or unique genotypes in 25% of Cypriot CF patients primarily due to the utilization of massively parallel sequencing and MLPA demonstrating their diagnostic robustness and utility by achieving a population specific detection rate of 96.3% for the entire patient population listed in the national registry.

In agreement with our previous report [5], the indigenous variant p.Leu346Pro (L346P), which is encountered only in individuals of Cypriot descent, is the second most common mutation (6.5%), detected in seven compound heterozygous cases. The p.Leu346Pro (L346P) missense mutation is generally associated with a milder disease phenotype, i.e. dominating over more severe mutations in compound heterozygous cases [9]. Nevertheless, the seven patients bearing this mutation demonstrated significant phenotypic variability, which could be attributed to ion channel disorders affecting CFTR function [25, 26], or other comorbidities [27–29]. A profound example is the severe case (see Additional file 1) with Huntington’s comorbidity and motor symptoms [30] that could have contributed to deterioration of chronic lung disease, underlining the possibility of combined effects of CF with concurrent other rare genetic diseases affecting the respiratory system.

The c.489 + 3A > G (621 + 3A > G) splicing mutation was identified *in cis* with p.Cys1400X (4326delTC) mutation in three compound heterozygous young patients with moderate clinical phenotype. Interestingly, co-segregation of these *CFTR* variants was previously reported in an Algerian patient [31] and possibly in four Greek patients [14, 32]. The c.489 + 3A > G mutation was initially considered as a severe mutation affecting the splicing of *CFTR* transcripts, reducing the amounts of normal mRNA and functional protein, and eventually leading to a severe disease phenotype [32]. However, its pathogenicity was later disputed based on more advanced epidemiological and molecular evidence [14], as it was found to be frequent among healthy individuals, and associated with the production of adequate amounts of correctly spliced mRNA and functional protein, despite its putative effect on *CFTR* transcript processing. The severe disease phenotype previously described in four Greek patients was probably attributed to another undetected mutation, possibly p.Cys1400X, which is commonly co-inherited with c.489 + 3A > G in cis as a complex allele. The cluster of complex alleles where c.489 + 3A > G co-segregates in cis with p.Cys1400X in Eastern Mediterranean and North Africa indicates that it is likely of common origin in the region.

Rearrangements within the *CFTR* gene, including large duplications and deletions, account according to CFTR1 for approximately 2% of the > 2000 known *CFTR* variants, although their frequency in specific ethnic groups has not been comprehensively and systematically assessed thus far. Interestingly, two large rearrangements, novel CFTR-dup2 found by us and the Slavic large deletion c.54-5940_273 + 10250del21kb (CFTRdele2,3(21 kb)) cumulatively account for 7.4% of all mutations in Cypriot CF patients. The frequency of this rare group of *CFTR* mutations is significantly higher than in other Mediterranean CF populations, such as the Italians [33] and Spaniards [34], where it is reported to be roughly 2.5% and 1.3%, respectively. The rare CFTR-dup2 mutation was previously reported to cause a mild disease phenotype when in trans with another common mutation [35]. However, CFTR-dup2 homozygosity is reported for the first time in our two cases. These patients were characterized by hepatobiliary disease, which in one of them was severe eventually leading to lethal liver failure, indicating CFTR-dup2 association with a more severe CF phenotype. Further data are warranted for the determination of the exact phenotype and appropriate treatment approaches in patients bearing this rare allele. Based on our experience, we recommend that in patients bearing two CFTR-dup2 mutations, prophylactic administration of ursodeoxycholic acid is started at an early age, and hepatic function is regularly assessed.

The *CFTR* variant p.Ser877Ala is thus far considered variant of uncertain significance. However, in compound heterozygosity with a common mutation in a young patient from Cyprus, p.Ser877Ala was associated with normal sweat chloride levels, normal lung function, but also with chronic respiratory symptoms, mild bronchiectatic changes, and recurrent *Pseudomonas aeruginosa* isolation in sputa cultures. Based on these observations, p.Ser877Ala could potentially be associated with CFTR-related diseases, when *in trans* with another CF-causing mutation.

The classification of individuals with sweat chlorides < 60 mmol/L is a grey area and depends on the definition used for CFTR-RD, especially when they bear novel or rare genotypic alterations of varying clinical consequence. In this study, we have used the Bombieri et al. [16] definition for the classification of marginal cases as CFTR-RD, but should we have used other definitions by Farrell et al. [36] or Castellani et al. [37], classification of a couple of cases would have been different. Ambiguity in the classification of individuals with mutations of varying clinical consequence inherited in compound heterozygosity with p.Phe508del or other confirmed pathogenic mutations may also have important implications in the era of precision medicine and the use of expensive CFTR modulators.

In terms of population genetics, the spectrum of non-p.Phe508del mutations observed in Cypriot CF population is generally distinct from neighbouring CF populations in countries surrounding the Eastern Mediterranean basin. However, some of these mutations observed at lower frequencies in Cypriots are relatively more common in Turkish (1677delTA, G542X and 2183AA > G) [20], Israeli Ashkenazi Jewish (W1282X, N1303K, G542X, 3849 + 10kbC > T) [38], Lebanese (N1303K, W1282X, S549N) [39], Greek (621 + 1G > T, G542X, N1303, 2789 + 5G > A, 2183AA > G), and Egyptian (2183AA > G, N1303K, W1282X, G155D, CFTRdele23 (21 kb) [40] CF populations in decreasing order of their relative frequencies. In fact, many of the *CFTR* rare alleles were also previously detected (as per CFTR1 database) in other CF populations residing around the Mediterranean basin, such as Q1476X (in Tunisia) [41], R1066C (in Southern Italy) [42], 4382delA (in Southern France) [43] and E379X (in Greece) [44], or reported in a Greek-Cypriot patient (M348K) [45].

## Conclusions

In this study, we demonstrated that the contribution of CF patient registries is particularly important in small or isolated populations, such as in Cyprus, where unique genotypic (e.g. documented founder effect in p.Leu346Pro (L346P), presence of complex allele p.Cys1400X with c.489 + 3A > G in cis, relatively common intra-*CFTR* rearrangement CFTR-dup2, including to a large degree a distinct mutation spectrum from neighbouring populations in the Eastern Mediterranean basin) and particular phenotypic profiles can be found. These observations together with comorbidity with other common recessive disorders in the region shed light on thus far less explored aspects of CF in specific populations. This study also corroborates the utility of massively parallel sequencing of the *CFTR* locus together with the use of the MLPA technique in terms of robust and accurate ascertainment of common and rare variants. The recent establishment of a national patient registry in the country has documented similarities between local CF population with other European countries, but also several notable distinct features, which are attributed to either regional environmental factors, such as the warm climate, or impact of rare *CFTR* variants on the course of CF. Finally, our data provide a strong basis for the improvement of CF genetic diagnostics, the eventual introduction of a multi-tier strategy using *CFTR* genotyping, and the introduction of CFTR-modulator therapies in the Cypriot CF population.

## Methods

### National CF patient registry

The national CF patient registry in Cyprus was established in 2017 based on the operating procedures and definitions of the ECFSPR. Initial approval from the Cyprus National Bioethics Committee (EEBK ΕΠ 2017.01.117) was limited to retrospective collection of demographic, clinical and laboratory data. Subsequent approval covered prospective data collection and CFTR genotyping. A written informed consent was provided by the patients or their guardians. To protect patient confidentiality, pseudonymisation was used for their identification within the collaborative genetic testing scheme*.*

### Patient selection

The patient status was defined according to the diagnostic inclusion criteria of the ECFSPR [17]: (a) two sweat chloride test values of at least 60 mmol/L, or (b) one sweat chloride test value of at least 60 mmol/L and two disease-causing *CFTR* mutations, or (c) typical CF features at clinical presentation and two disease-causing *CFTR* mutations if sweat chloride test value was less than 60 mmol/L or not reported. Patients bearing one CF mutation and one CFTR-related disease mutation or two CFTR-related disease mutations and a sweat chloride of less than 60 mmol/L were classified as CFTR-related disease [16].

Genotyping was performed in all of our patients, whereas most of them underwent quantitative measurement of sweat chloride with the pilocarpine iontophoresis stimulation of a localized skin area [8] method. The earliest date when at least one of the above criteria was fulfilled was considered as the date of diagnosis.

### Sanger DNA sequencing/fragment analysis and CFTR genotyping (Cyprus)

Genomic DNA was isolated from peripheral blood leukocytes using the QIAamp Blood Midi Kit™ (QIAGEN.com, GmbH D-40724, Hilden, Germany). We have utilized a ‘cascade cost saving approach’ by initially testing for the most common mutation p.Phe508del (F508del) by amplifying a 79 bp DNA fragment containing exon 11 of the *CFTR* gene with the following primers: CF10 forward 5′-GTT TTC CTG GAT TAT GCC TGG C-3′, and CF10 reverse 5′-GTT GGC ATG CTT TGA CGC TTC-3′. The 20 µL PCR reaction mixture contained 100–200 ng of genomic DNA, 1 × PCR buffer, 200 µM of each dNTP, 5 pmol of each primer and 0.5 unit of AmpliTaq Gold™. The reaction mixture was subjected to one cycle of denaturation at 95 °C for 5 min followed by 35 cycles of denaturation at 94 °C for 1 min, annealing at 58 °C for 1 min, extension at 68 °C for 1 min and a final extension at 68 °C for 6 min. An aliquot of the PCR product was subjected to cycle sequencing using the BigDye Termimator™ and was electrophoresed and sized on the automated ABI 3130xl Genetic Analyser™ (all supplied Thermo Fisher Scientific; USA www.thermofisher.com). Subsequently, in cases where one or both CF alleles remained unidentified, we initially used the ElucigeneCF29 v.2™ assay. This assay has been replaced since 2019 with its more advanced ElucigeneCF-EU2v1™ version (both Elucigene Diagnostics, United Kingdom; www.elucigene.com) that has higher non-p.Phe508del (F508del) detection rate in our geographic region.

### MLPA-based intra-CFTR rearrangement analysis (Cyprus and Czechia)

DNA from all individuals in this study was also examined. MLPA was employed to investigate any possible large intra-*CFTR* rearrangements using probe mixture P091-D2 *CFTR* according to the manufacturer’s recommendation. The Coffalyser.net software was used for graphical and statistical analyses (MRC. Holland, www.mlpa.com).

### Massively parallel sequencing of the CFTR locus (Czechia)

Analysis of the entire *CFTR* coding region, adjacent splice site junctions and several introns using a locus-specific library preparation assay (CFTR NGS assay™; Devyser, Sweden) while massively parallel sequencing was performed on the MiSeq System™ in 12 cases (Illumina; USA). Bioinformatic analysis was carried out using the SOPHiA Platform for Hereditary Disorders™ (SophiaGenetics; Switzerland). Positive cases were confirmed by targeted Sanger DNA sequencing on ABI 3130xl DNA Analyser™ (ThermoFisher Scientific; USA). MLPA was carried out in the same manner as listed above on a selected subset of patients not tested by this methodology in Cyprus. Where applicable (e.g. in further discussed complex *CFTR* alleles with two variants *in cis*), the linkage phase of detected mutations was confirmed by their re-analysis in patients’ parents.

### CFTR variant nomenclature and classification of CF-causing mutations

For the *CFTR* variant nomenclature we used the recommendation of the Cystic Fibrosis Mutation Database (CFTR1; Toronto, Canada; www.gene.sickkids.on.ca). However, in particular in the Discussion section we resorted to the still more widely understood ‘legacy nomenclature’ indicated in parentheses. Variant pathogenicity was assessed according to the ‘Clinical and Functional Translation of CFTR’ database (CFTR2; Baltimore, USA; www.cftr2.org) where applicable.

### Demographic and clinical data

Patients were categorized into three main groups: (a) living patients who were seen by a CF specialist at least once during the last 3 years (2017–2019), (b) patients who were lost to follow-up, i.e. not seen for more than three consecutive years, and (c) deceased patients.

Patients’ data were distinguished to (a) baseline data, including demographics, age and clinical presentation at diagnosis, sweat chloride concentrations (in mmol/L), and *CFTR* genotype, and (b) annual follow-up data from both scheduled clinical visits and hospital admissions during the last 3 years, including clinical manifestations, anthropometrics, spirometry, airway microbiology, medical imaging, multi-systemic complications, and treatment modalities. For the purpose of this study, annual follow-up data from the last completed year, i.e. 2019, were used, while most recent data from 2018 were used for the cases that had not attended the CF clinic during 2019. Patients’ age at follow up is generally reported as of December 31, 2019.

Clinical data were retrieved from patients’ medical records. Spirometry (Vitalograph Pneumotrac, Vitalograph Inc.; USA) was routinely performed in every clinical visit in patients above 6 years of age, according to the American Thoracic Society (ATS) and European Respiratory Society (ERS) guidelines (Graham et al., 2019). The best FEV_1_ and FVC values per year were recorded. The z-scores for BMI and FEV_1_ were calculated using the WHO AnthroPlus® (WHO, 2009) and the GLI Online Calculator® (Quanjer et al., 2012) software systems, respectively. Sputum or cough swab microbiology testing was performed routinely in patients during each clinical follow-up. Chronic lung colonization with *Pseudomonas aeruginosa* was defined according to the modified Leeds criteria, i.e. when more than 50% of the cultures were positive for the pathogen, provided at least 4 cultures were performed in the previous year (Zolin et al., 2019). High-resolution chest computed tomography (HRCT) scans were performed in the majority of patients.

### Statistical methods

Categorical variables are presented as frequencies (%), while continuous variables are presented as mean (standard deviation). Categorical comparisons were calculated using the chi-squared test. All summary statistics and statistical comparisons were calculated using STATA 12 (Version 12, StataCorp, College Station; USA).

## Supplementary Information

**Additional file 1**. Detailed Description of Methods and Rare Cases.

## Data Availability

The data that support the findings of this study are available from the Cyprus National Cystic Fibrosis Patient Registry, but restrictions apply to the availability of these data, which were used under license for the current study, and so are not publicly available. Data are however available from the authors upon reasonable request and with permission of the cystic fibrosis patients participating in the Registry.

## Abbreviations

ATS: American Thoracic Society
BMI: Body mass index
CF: Cystic fibrosis
CFTR: Cystic fibrosis transmembrane conductance regulator
DNA: Deoxyribonucleic acid
dNTP: Deoxynucleoside triphosphate
ECFSPR: European Cystic Fibrosis Society Patient Registry
ERS: European Respiratory Society
FEV_1_: Forced expiratory volume in 1 s
FVC: Forced vital capacity
GLI: Global lung function initiative
HRCT: High-resolution computed tomography
MIM: Mendelian inheritance in man
MLPA: Multiplex ligation-dependent probe amplification
PCR: Polymerase chain reaction
WHO: World Health Organisation
