# Supplementary material for: Demographic characteristics, clinical and laboratory features, and the distribution of pathogenic variants in the CFTR gene in the Cypriot cystic fibrosis (CF) population demonstrate the utility of a national CF patient registry
Source: Orphanet J Rare Dis. 2021 Oct 2;16:409. doi: 10.1186/s13023-021-02049-z (PMC8487500; doi:10.1186/s13023-021-02049-z)
Supplement: Supplementary file 1 — Additional file 1. Detailed Description of Methods and Rare Cases. [file 13023_2021_2049_MOESM1_ESM.docx]

**Table S3. Treatment modalities in 2019 or 2018**

| **Treatment modality** | **Number of patients** | **Percentage of patients (%)** |
| --- | --- | --- |
| ***Respiratory*** | | |
| Continuous inhaled hypertonic saline | 5 | 16.7 |
| Continuous inhaled mannitol | 0 | 0 |
| Continuous inhaled antibiotics | 9 | 30 |
| Continuous inhaled bronchodilators | 13 | 43.3 |
| Oxygen therapy | 0 | 0 |
| Continuous NIPPV* | 0 | 0 |
| Recombinant human DNAse | 19 | 63.3 |
| Continuous inhaled steroids | 11 | 36.7 |
| Continuous oral steroids | 0 | 0 |
| Continuous azithromycin or other macrolides | 12 | 40 |
| Chest physiotherapy | 16 | 53.3 |
| ***Gastrointestinal, hepatobiliary and pancreatic substitution*** | | |
| Ursodeoxycholic acid | 2 | 6.7 |
| Pancreatic enzymes | 20 | 66.7 |
| Proton pump inhibitors | 2 | 6.7 |
| Caloric fortification | 6 | 23.3 |
| Multivitamins | 23 | 76.7 |
| Electrolyte solutions | 2 | 6.7 |
| ***Targeted therapy*** | | |
| CFTR modulators | 3 | 10 |

**Legend:** 2018 data were used in cases which did not attend the CF centre during 2019; *NIPPV: Non-invasive positive pressure ventilation.

**Detailed presentation of notable rare or unique cases cases from the Cyprus National CF registry**

**Cases with the p.Leu346Pro (L346P) mutation in compound heterozygosity**

The p.Leu346Pro (L346P) mutation has been reported only in patients of Cypriot descent. It was identified in compound heterozygosity in seven patients, usually presenting in childhood with dehydration or electrolyte imbalance, followed by late-onset lung disease and bronchiectasis in adolescence and early adulthood. A 48-year female patient, who also has Huntington’s disease (MIM: 143100), demonstrated severe lung disease. The patient had chronic Pseudomonas aeruginosa airway colonization, very low lung function z-scores (FEV1=-4.7 and FVC=-3.3), and diffuse severe bronchiectasis in the right lung, atelectatic left lung with multiple bullae and ipsilateral mediastinal shift.

**Cases with the complex allele p.Cys1400X with c.489+3A>G in cis**

Three young patients, a 1-year female and two 9- and 18-year males, were identified with the known p.Cys1400X (4326delTC) in compound heterozygosity with another known CFTR mutation. In all three cases, as shown by parental genetic testing, p.Cys1400Ter co-segregates with the rare splicing mutation c.489+3A>G (621+3A>G), which was originally detected in Greece and previously reported as having varying clinical consequence [1]. According to the CFTR2 database data, it is more likely to be associated with pancreatic sufficiency ([www.cftr2.org/mutation/general/621%252B3A-%253EG/](http://www.cftr2.org/mutation/general/621%252B3A-%253EG/)). The first case was diagnosed through neonatal screening when the index case´s family lived in the United Kingdom, while the other two cases presented in early childhood with respiratory symptoms and electrolyte imbalance. Spirometry in these two patients showed a mean best FEV1 z-score of -1.8 and a mean FVC z-score of -0.9, while chest imaging revealed extensive bronchiectatic changes. The mutations in the other CFTR allele in these three cases are a large rearrangement c.54-5940_273+10250del21kb (CFTRdele2,3), the novel p.Gly178TrpfsX5 (c.531dupT), and the common p.Asn1303Lys (N1303K), respectively. In addition, one of the cases was found to have a benign intronic CFTR variant c.3469-65C>A (3601-65C/A), found previously in disseminated bronchiectasis, which is co-segregating with N1303K in cis.

**Cases with homozygosity of the CFTR-dup2 intra-CFTR rearrangement**

Two adult male siblings of Greek-Cypriot origin were found to bear a novel duplication of exon 2 on both parental CFTR alleles detected by massively parallel sequencing and confirmed independently by the MLPA technique. Although patients’ parents come from the same small village, we could not ascertain parental consanguinity. The first case presented with persistent respiratory symptoms, pancreatic insufficiency and malnutrition at the age of 41 years and was diagnosed by two positive sweat chloride tests (75 and 117 mmol/L) and subsequent massively parallel sequencing revealed CFTR-dup2 in homozygosity. Patient had chronic Pseudomonas aeruginosa pulmonary colonization and mild bronchiectatic changes on chest HRCT scan. In 2019, his best FEV1 and FVC z-scores were -0.7 and 0.1, respectively, with a BMI z-score of -0.1. However, the patient developed severe hepatic involvement with biliary cirrhosis and eventually hepatic failure that led to his death at the age of 47 years.

The second case was diagnosed by a positive sweat chloride test (85 mmol/L) and identification of CFTR-dup2 mutation in homozygosity on massively parallel sequencing at the age of 49 years. The patient reported persistent respiratory and gastrointestinal symptoms since childhood. He was chronically colonized with Pseudomonas aeruginosa with moderate bronchiectatic changes and lung emphysema on chest CT scan in the presence of digital clubbing. He was a tobacco smoker for years in the past and his spirometric indices were extremely low with best FEV1 z-score of -5.2, FVC z-score of -0.5, and BMI z-score of 0.3. Abdominal ultrasonography showed an enlarged and diffusely hyperechogenic liver, although liver function tests are to date within normal range. Increased plasma amylase and fasting glucose levels on serial measurements were indicative for chronic pancreatitis and CF-related diabetes mellitus, respectively.

**Cases with compound heterozygosity of the novel p.Ser877Ala variant**

A 16-year old male was diagnosed at the age of 14 years after presenting with persistent respiratory symptoms. He is compound heterozygous for the common p.Gly542X (G542X) mutation and the novel CFTR variant p.Ser877Ala and had two negative sweat chloride tests (24 and 21 mmol/L). According to the ACMG.net classification this variant is Class 3 – variant of unknown significance (Harrison et al., 2019). His best FEV1 z-score was 0.2, and FVC z-score -0.8, and he is a high-performing athlete. Although several sputum cultures were positive for Pseudomonas aeruginosa, the modified Leeds criteria for chronic colonization were not fulfilled. Chest HRCT scan revealed localized mild bronchiectatic changes. Therefore, this case is classified as a CFTR-related [2] disorder and the patient will be continuously monitored for the eventual development of CF symptoms in adulthood.

**References**

1. Forzan M, Salviati L, Pertegato V, Casarin A, Bruson A, Trevisson E, Di Gianantonio E, Clementi M: Is CFTR 621 3 A> G a cystic fibrosis causing mutation? J Hum Genet 2010, 55(1):23-26.
2. Bombieri C, Claustres M, De Boeck K, Derichs N, Dodge J, Girodon E, Sermet I, Schwarz M, Tzetis M, Wilschanski M: Recommendations for the classification of diseases as CFTR-related disorders. Journal of Cystic Fibrosis 2011, 10:S86-S102.
